# The Co-infection of Mild COVID-19 and Rhinocerebral Mucormycosis in a Patient Without Diabetes or Prior Steroid Use

**DOI:** 10.7759/cureus.24986

**Published:** 2022-05-14

**Authors:** Nishanth Sekar, K T Sundaresan

**Affiliations:** 1 Internal Medicine, Teaching Hospital Batticaloa, Batticaloa, LKA; 2 Clinical Sciences, Eastern University of Sri Lanka, Batticaloa, LKA

**Keywords:** antifungal drugs, diabetes type 2, steroid use, covid 19, rhinocerebral mucormycosis

## Abstract

In a fast-evolving COVID-19 pandemic, co-infection with mucormycosis has been reported in some parts of the world. It is still unknown whether one of the either diseases makes the patient susceptible to developing the other. The co-occurrence of them significantly elevates the mortality risk and is commonly reported in immunocompromised individuals. We herein report a case of COVID-19 infection with rhinocerebral mucormycosis without prior steroid use or underlying immunosuppressive diseases.

## Introduction

There are a few cases of COVID-19 infection and associated mucormycosis that are described around the world. But most of the co-infections are attributed to steroid use that is related to the COVID-19 infection or any underlying immunosuppressive conditions like diabetes mellitus [1].

However, some susceptible patients can develop them as co-infection without any identifiable risk factors. Therefore, the prime goal is to have high clinical suspicion and diagnose them as quickly as possible and manage them with the inputs from the multidisciplinary team appropriately.

There are several types of mucormycosis infection, namely the cutaneous, rhinocerebral, pulmonary, gastrointestinal and disseminated variants. The rhinocerebral variant that occurs nearly in half of the affected patients starts in the nose and eventually reaches the orbital cavity and central nervous system [2].

Aggressive treatment with intravenous antifungals needs to be started as soon as possible for a good outcome [2]. Due to the destructive nature of the disease, complete recovery sometimes needs repetitive surgical interventions.

## Case presentation

**Figure 1:**
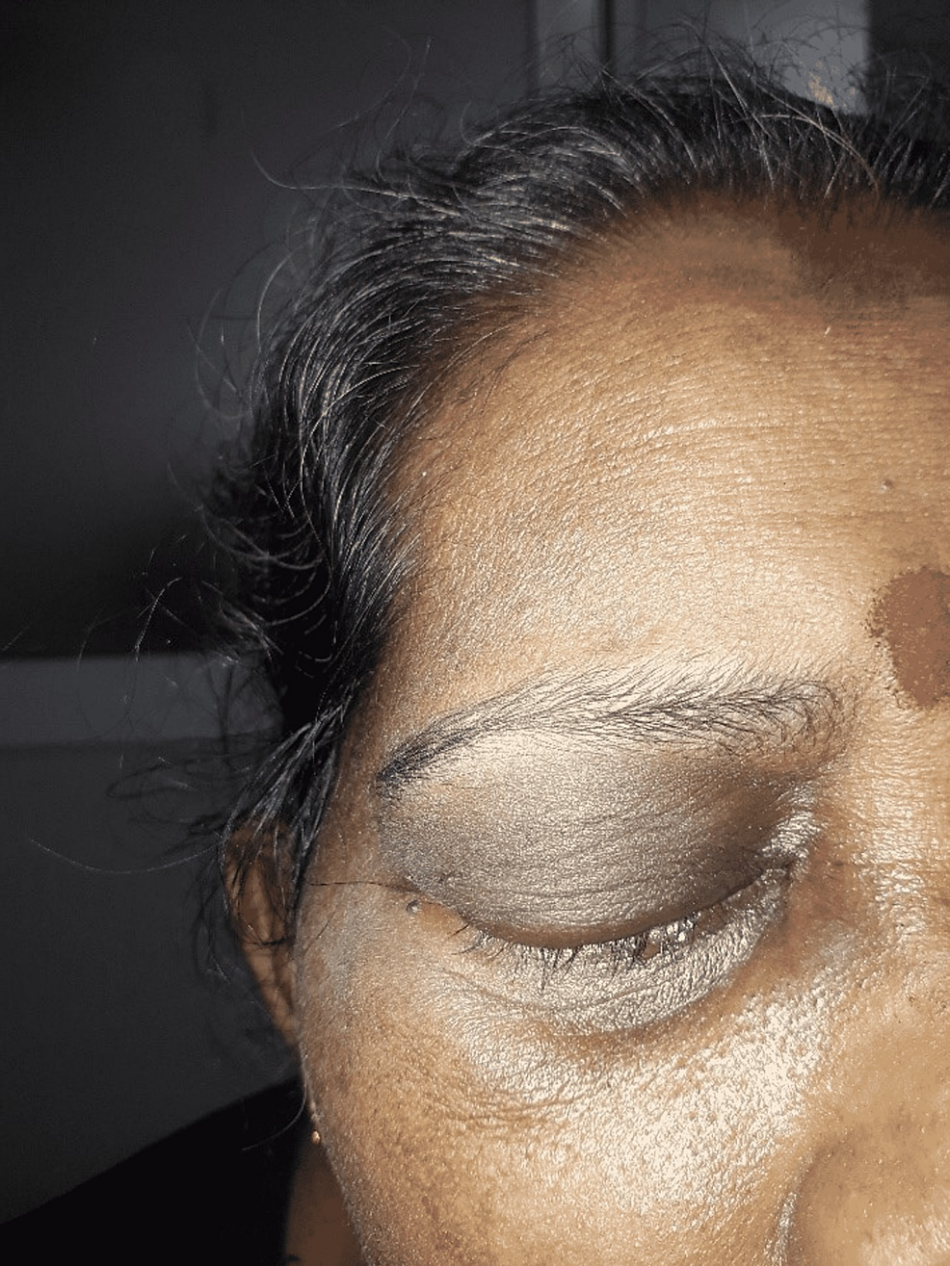
The patient with the periorbital discoloration and complete ptosis

A 59-year-old South Asian female patient with no premorbid conditions (unremarkable past medical history) presented with fever, productive cough and right-sided periorbital swelling and complete drooping of the right eyelid with discoloration for the one-week duration (Figure 1). Right-sided vision has become gradually obscured over the days. She denied any shortness of breath upon exertion or at night. She had no urinary symptoms and the bowel habit was unaltered.

On examination, she was febrile, but not pale or icteric. There was no lymphadenopathy. Neck stiffness and Kernig’s sign were not detected. She had no peripheral stigmata of infective endocarditis. There was no pitting ankle edema. Neurology examination showed right-sided complete ptosis with third nerve palsy without pupillary involvement. The right-sided visual acuity was 6/12 with a preserved left-sided vision. The other cranial nerves (except the second and third cranial nerves) and upper and lower limbs were neurologically unaffected.

Cardiovascular examination showed a blood pressure value of 130/85 mmHg with a heart rate of 96 beats per minute which was regular and of good volume. There was no murmur. Respiratory system examination revealed a respiratory rate of 22 per minute and oxygen saturation of 96% on room air. The modified exertional desaturation test was negative. Chest proper examination was unremarkable except for a few occasional coarse crepitations. The abdomen examination was virtually normal. The following investigations were performed (Table 1).

**Table 1:** The set of investigations done CRP: C-reactive protein, ESR: Erythrocyte sedimentation rate, LDH: Lactate dehydrogenase, ALT: Alanine transaminase, AST: Aspartate transaminase, ALP: Alkaline phosphatase, NS1 antigen: Non- structural protein- 1 antigen, INR: International normalizing ratio and APTT: Activated partial thromboplastin time, UFR: Urine full report, hpf: High power field

Test	Reference values	Results on admission	Results after 3 days	Results after 1 week
Full blood count				
White cell counts (10^3^/ µL)	4-11	7.85	6.43	6.34
Neutrophils (10^3^/µL)	2-7	5.35	4.8	4.6
Lymphocytes (10^3^/µL)	1-5	2.1	1.4	1.24
Eosinophils (10^3^/µL)	< 0.5	0.16	0.1	0.2
Monocytes (10^3^/µL)	0.2- 0.8	0.24	0.13	0.3
Platelets (10^3^/µL)	150-400	245	165	198
Hemoglobin (g/dL)	11- 15	13.2	13	13.4
CRP (mg/L)	< 5	106	47	22
ESR (mm/hr)	< 22	57		20
LDH (U/L)	< 234	232		132
Serum ferritin (ng/mL)	10-120	210		75
Serum sodium (mmol/L)	135-145	138	136	141
Serum potassium (mmol/L)	3.5-5.1	4.1	3.7	4.2
Serum calcium (mmol/L)	2.1-2.6	2.3	2.5	2.4
Serum creatinine (µmol/L)	100-115	101	105	104
Blood urea (mmol/L)	3-7	6.5	6.7	6.4
ALT (U/L)	10- 50	34	36	27
AST (U/L)	10- 40	22	26	24
ALP (U/L)	25-150	46	48	40
Gamma- glutamyl transferase (U/L)	10-65	43	40	54
Total protein (g/L)	65-83	64	62	66
Serum albumin (g/L)	35-50	36	34	36
Serum globulin (g/L)	20-40	28	28	30
Total Bilirubin (µmol/L)	5-17	9	9.8	10.2
INR	< 1.1	0.5		
APTT	30-40 seconds	27		
COVID-19 polymerase chain reaction		Positive		
NS1 antigen		Negative		
Rapid antigen test		Positive		
UFR: Pus cells	Nil	Nil		
UFR: Red cells	Nil	1-2/hpf		
UFR: Albumin	Nil	Nil		
Blood culture		No growth		
Urine culture		No growth		
Blood picture		Normal		
Chest x-ray		Patchy inflammatory shadow		
Electrocardiogram		Sinus rhythm		
2D echocardiography		Ejection fraction: 60% with normal valves		
Ultrasound scan of the abdomen		Normal kidneys No hepatosplenomegaly or lymphadenopathy		

**Figure 2:**
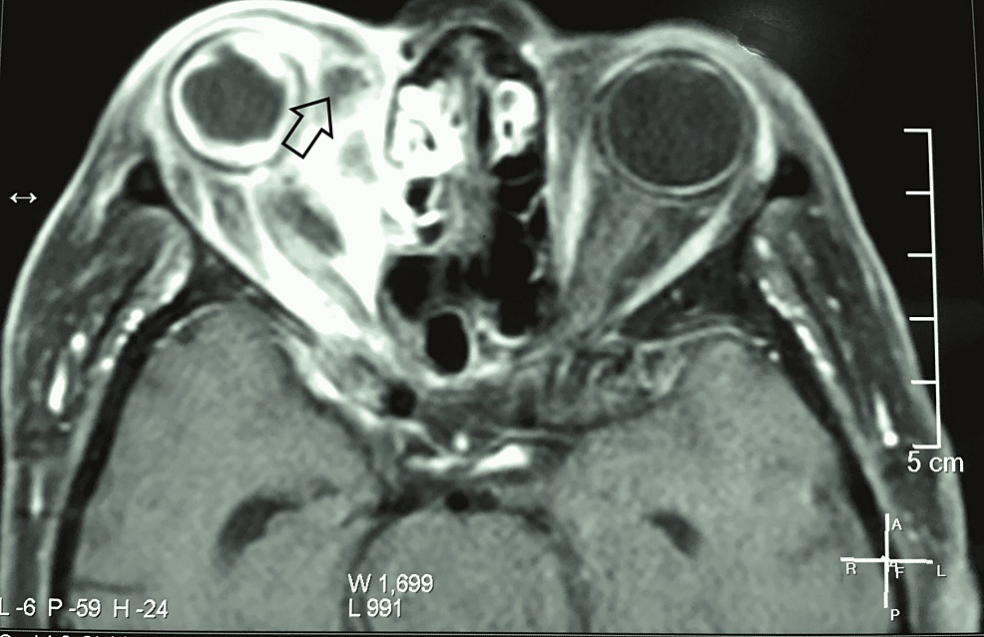
Magnetic resonance imaging of the brain and paranasal sinuses showing right-sided periosteal abscess formation

**Figure 3:**
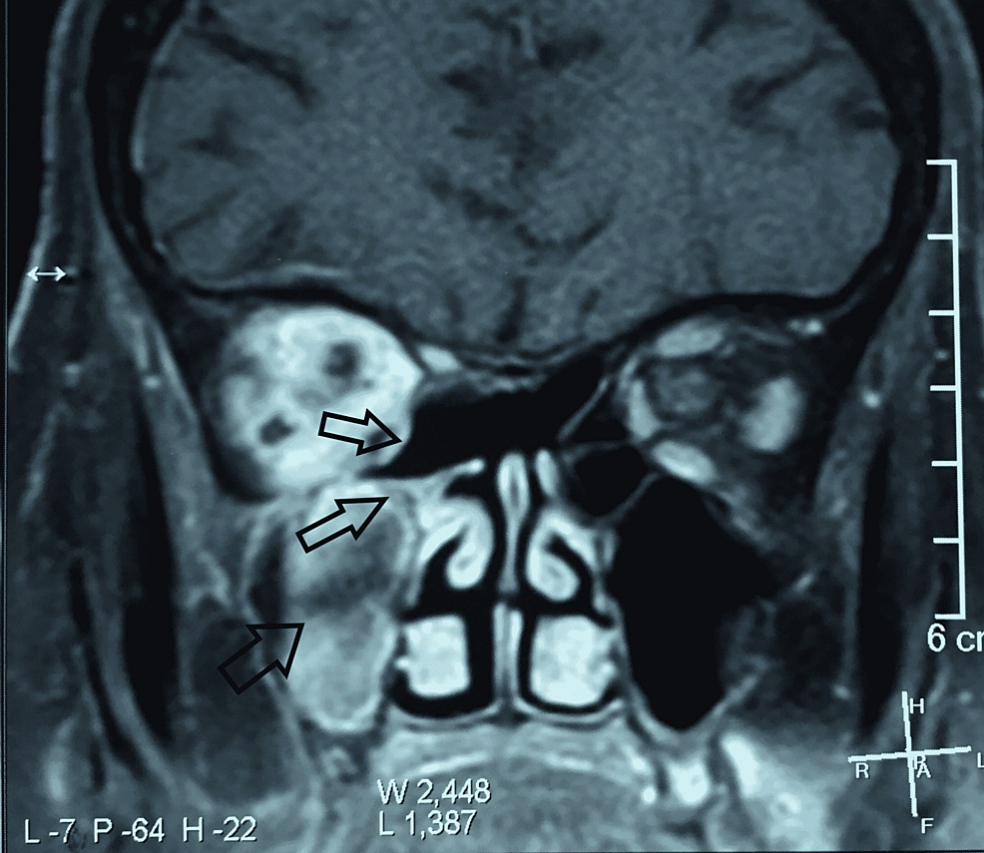
Magnetic resonance imaging of the sinuses showing right-sided frontal, maxillary and ethmoidal sinusitis

The magnetic resonance imaging of the brain and sinuses was done to evaluate the extent of the involvement. It revealed right-sided orbital subperiosteal abscess formation (Figure 2) with evidence of right-sided frontal, maxillary and ethmoidal sinusitis (Figure 3).

 

**Figure 4:**
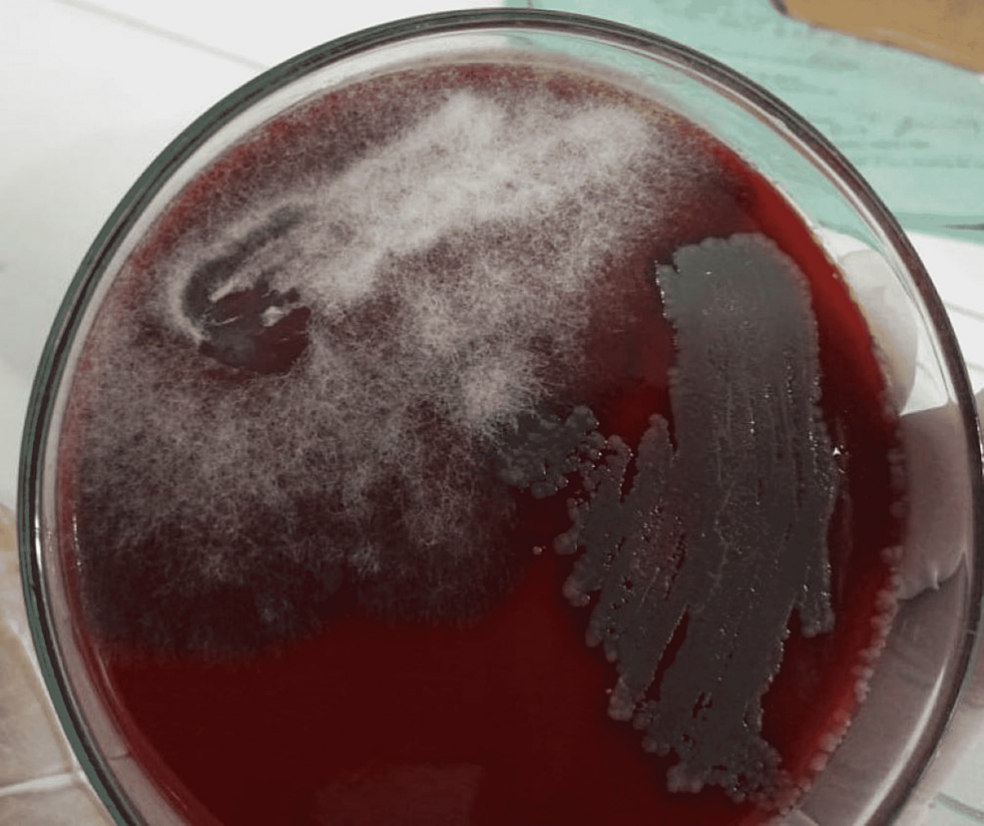
Sheep blood agar (SBA) showing colonies of filamentous fungal growth

Ear, nose and throat team opted to perform the rigid nasal endoscopy from which residual sinus secretion was obtained. It was incubated on the Sheep blood agar at 37 degree celsius for 48 hours, which showed multiple colonies of filamentous fungal growth (Figure 4).

**Figure 5:**
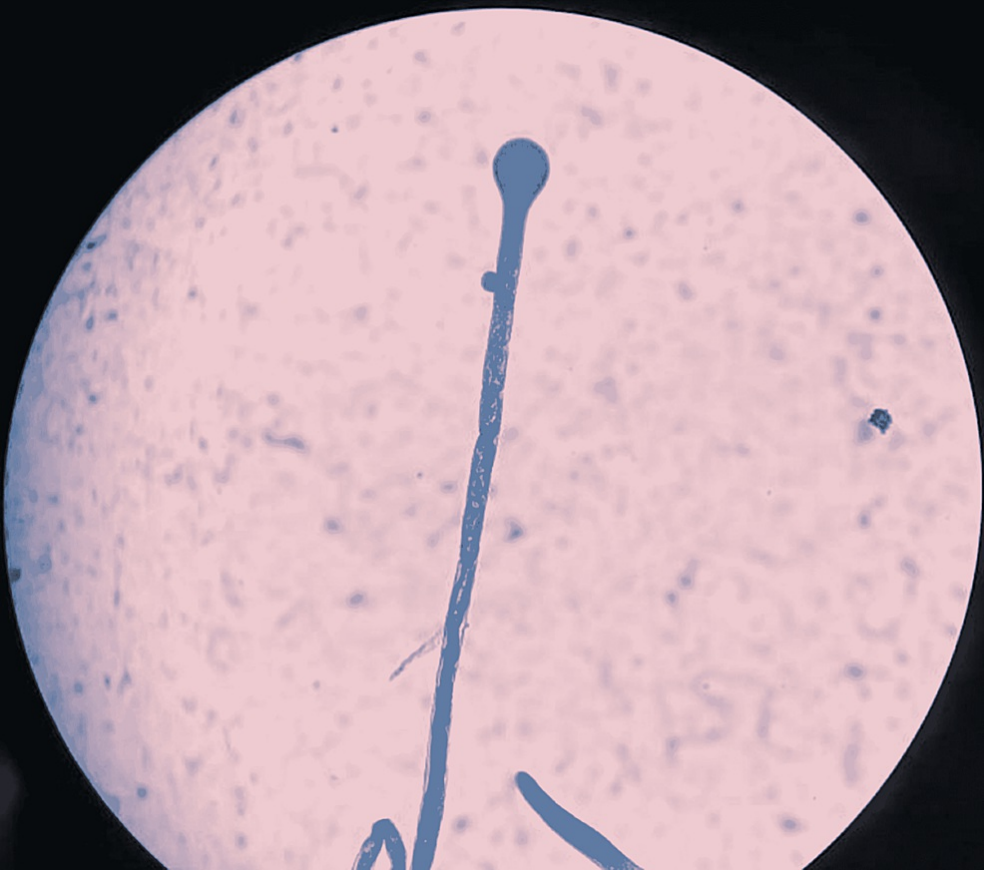
Microscopy of the growth under light microscope (40 x 10) showing broad non-segmented hyphae

Microscopy of the growth under light microscope (40 x 10) revealed broad non-segmented hyphae suggestive of mucormycosis (Figure 5). The culture was sent to the reference laboratory and was confirmed as mucormycosis species.

She was admitted to the COVID-19 isolation unit. Her fasting blood sugar and the capillary blood sugar readings remained within normal range throughout. With the clinical suspicion, we started intravenous Liposomal Amphotericin 3 mg/kg/ day before the receival of the biopsy report. A multidisciplinary team meeting was arranged encompassing the consultant physician, consultant ear, nose and throat surgeon, consultant microbiologist and consultant surgeon to arrive at a comprehensive management plan. For the mild COVID-19, considering a secondary bacterial infection (as evidenced by the patchy inflammatory shadow on the chest x-ray), we started Intravenous Ceftriaxone 1 gram twice daily and oral Clarithromycin 500 mg twice daily with the multidisciplinary consensus.

The patient was well-hydrated and the renal function was monitored every other day considering the nephrotoxic nature of the antifungal therapy. On day 10, patient’s COVID-19 polymerase chain reaction turned negative and the patient was taken out of the COVID-19 isolation unit. The antibiotic were omitted at the end of two weeks. After four weeks of intravenous Amphotericin, the fungal culture was repeated from the maxillary sinus which was negative for fungal hyphae. Patient’s periorbital swelling improved and inflammatory markers returned to normal values. The patient underwent surgical debridement twice by the ear, nose and throat team during the admission to clear the residual tissue. She was discharged after one month of admission with a comprehensive follow- up plan.

## Discussion

COVID-19 infection is categorized into mild, moderate and severe forms according to the severity of the illness. The common clinical presentations include fever, cough and shortness of breath. Supplemental oxygen and corticosteroids are the key aspects of the treatment protocol for COVID-19 [3]. COVID-19 patients with persistent hyperglycemia are in the risk zone of developing mucormycosis.

Although the main risk factors for the development of mucormycosis are touted to be excessive antibiotic use, irrational steroid use, diabetes and hypoxia, environmental factors may play a significant role in enhancing the disease progression [4].

This exciting manuscript has described that that is not always the case. Some instances of coexisting COVID-19 infection and mucormycosis have no underlying etiological basis.

Mucormycosis is an acute fungal infection caused by the members of mucoraceae family [5]. Rhizophus species are the most common causative agents in this group. It can occur as an opportunistic and fatal fungal disease in immunocompromised patients such as those with diabetes. The most common clinical form is the rhino-orbito-cerebral variant [5]. Before the development of the pulmonary and disseminated manifestations, mucormycosis commonly begins as the rhino cerebral form. Infection begins in the nasal cavities and paranasal sinuses and progresses to its full form. The common presentations are facial swelling, headache, fever, eyelid drooping and black lesions on the periorbital and nasal region. Infarction and necrosis of host tissues occur due to the invasion of non-septate hyphae. The diagnostic tests are mainly histopathology, direct testing, and culture of clinical specimens [5]. The treatment option is intravenous liposomal Amphotericin [5]. The therapy must continue until the patient makes a complete clinical and radiographic resolution [6].

In our patient with an unremarkable past medical history, we established a diagnosis of mild COVID-19 infection and rhinocebreal mucormycosis. With the high clinical suspicion, we started the intravenous antifungals early in the treatment and liaised with the ear, nose and throat team for the surgical debridement of the necrotic tissue. We followed a multidisciplinary team approach which was so efficient and the patient made a complete recovery after a course of four weeks. We stopped the antifungal therapy after one month as the patient made a convincing clinical and radiographic resolution.

Mucormycosis is a life-threatening disease and the association with COVID-19 incurs a worse prognosis. Though the glucocorticoid therapy can mount a response against the interleukin-6 mediated cytokine storm in COVID-19, its overuse has predisposed to the development of mucormycosis. Especially in South Asian countries such as India with a high population, steroid use is strongly associated with mucormycosis [7].

In literature, it is the first reported case of mucormycosis and COVID-19 without steroid use or diabetes in the world. In nondiabetic individuals, several case reports describe that steroid use and immune dysregulatory mechanisms in COVID-19 are the main contributory factors for the co-infection and most of such cases are reported in the South East Asia [8]. Some case reports from India suggest that steroid usage during COVID-19 transiently causes hyperglycemia due to which the already immunocompetent individuals get the co-infections [9].

Some case reports accounting the aforementioned co-infection in the immunocompetent individuals suggest severe COVID-19-induced lymphopenia, T-cell dysregulation and other cytokine dysregulation as the probable causes for such a rare association [10].

There are several proposed reasons behind the development of co-infection in an immunocompetent individual. The repeated steaming during the COVID-19 era suppresses the nasal tract's advantageous microbiome and nasopharynx associated lymphoid barrier thereby favoring the entry of the fungus via the nasal route [11]. Dysbiosis of gut microflora has been caused by the abundant use of zinc supplementation during the COVID-19 times, providing a favorable environment for fungal growth [11]. Via the gut-lung axis, mucorale specific T cells (the CD4+ and CD8+ cells) interact with the gut microflora, which determines the overall functionality of the immune system, it's therefore important to maintain the gut microbiome intact. In addition, excessive vitamin A supplementation to get rid of COVID-19 may impair the humoral immunity (antibody-mediated) which in turn enables the fungal growth in our body [11].

The association is mostly observed during the hot and summer seasons with lower relative humidity in the tropical areas, raising the possibility of a seasonal trend [11]. However, the scientific data is lacking on this regard. More studies need to be done to evaluate the seasonal variation, ambient temperature and humidity and its relationship with the co-infection. The oxygen therapy during COVID-19 seems to have no impact on the development of mucormycosis [11]. Malnutrition related to COVID-19 may, nonetheless, play a role in the fungal invasion.

As there was a surge in the mucormycocis cases during the second COVID-19 wave especially in India, it was speculated that due to the shortage of oxygen supply systems, patients developed silent hypoxia (happy hypoxia). And thus, they experienced prolonged low oxygen levels ( <95%) and the hypoxia favored the virulence of the pathogenic fungi like mucormycosis [12]. Hypoxia is postulated to affect the transcriptional levels of genes associated with fungal virulence. Similarly, hypoxia induces the endocytosis of some mucorale species and shifts the source of fungal energy metabolism from carbohydrate to fatty acids, thereby using up lipid from the host serum [12]. This explains why the destructive lesions are more concentrated in the face area where the sebaceous glands are abundant. Furthermore, hypoxia inducible factor (HIF) causes tissue damage and immune malfunction [12].

In diabetic patients with COVID-19, strict glycemic control and careful steroid use is key to the prevention of mucormycosis [13]. And, it must be remembered that in non-diabetic patients with COVID-19, rational use of steroids is recommended to minimize the risk of mucormyscosis.

Treating mucormycosis is a multidisciplinary team approach which requires a set of radical and repetitive surgical debridement along with intravenous antifungals [14].

## Conclusions

The case strikingly describes a rare co-occurrence in an immunocompetent individual. The association between COVID-19 infection and mucormycosis can occur without any predisposing factors. We highlight the importance of having a high clinical suspicion and diagnosing them early so that antifungal therapy can be initiated as soon as possible. As both infections can assume a more devastating clinical course, it is highly recommended to start the definitive therapy early without waiting for the confirmatory investigations. Therapy must continue until the clinical and radiographic resolution is achieved. Apart from the antifungal therapy, several series of surgical debridement may be necessary for the complete cure due to the invasive nature of the mucormycosis.
